# Living and dying in dignity among older migrants

**DOI:** 10.1177/09697330261449309

**Published:** 2026-05-27

**Authors:** Anna Zhou, Ingrid Hellström, Susanne Roos, Åsa Larsson Ranada

**Affiliations:** 1 Department for Health, Medicine and Caring Sciences, HMV, 4566 Linköping University, Linköping, Sweden; 2 Department of Geriatrics, Local Health Care Eastern Östergötland in Vrinnevi Hospital, Norrköping, Sweden; 3 Department of Health Care Sciences, 7643 Marie Cederschiöld University, Stockholm, Sweden

**Keywords:** ageing, agency, belonging, communion, migration, person-centred care

## Abstract

**Background:**

Dignity is a foundational ethical principle in the care of older adults. Although widely discussed in nursing ethics, little is known about how older foreign-born adults themselves understand ageing with dignity, or how migration may shape this process in a welfare-state context.

**Aim:**

To explore how foreign-born older adults in Sweden perceive and articulate dignity while ageing.

**Research design:**

A Glaserian Grounded Theory approach guided data collection and analysis.

**Participants and research context:**

Five language-specific focus groups and one individual interview were conducted with 23 foreign-born adults aged 60 years and older living in Sweden.

**Ethical considerations:**

Ethical approval was granted by the Swedish Ethical Review Authority.

**Findings:**

The core category – *Living and Dying in Dignity*: *A Lifelong Negotiation of Control and Connection* – conceptualises dignity as an ongoing process shaped by balancing agency and communion. Four interrelated categories were identified: *The Prerequisites of Dignity*, in which selfhood, autonomy, and connection were foundational; *Maintaining Dignity in Everyday Life*, which captured strategies for preserving control, meaning, and continuity; *The Art of Letting Go*, involving adaptation, acceptance, and moral boundary-setting in the face of loss and suffering; and *Surrendering with Trust*, in which dignity was sustained through confidence in family, care systems, or existential frameworks when agency waned. Migration-related conditions, especially language, home, and cultural recognition, shaped how this negotiation unfolded.

**Conclusion:**

Dignity in later life was understood not as a fixed state but as a lifelong negotiation between control and connection. For older foreign-born adults, this negotiation was reconfigured by migration-related transitions, making recognition, home, and relational continuity especially significant.

## Introduction

Ageing with dignity is a central ethical ambition of eldercare systems worldwide and a fundamental human right.^1^ In Sweden, dignity is firmly embedded in health and social care legislation, which emphasises respect for equal human worth, personal integrity, and self-determination throughout later life.^2–4^

Like many European societies, Sweden is experiencing population ageing alongside the long-term demographic consequences of earlier migration. Approximately one-fifth of the population is foreign-born, reflecting decades of labour migration, refugee reception, and family reunification.^5^ As these cohorts grow older, increasing numbers of older adults are ageing within welfare and care systems shaped by social, institutional, and normative frameworks different from those of their earlier lives.

Ageing and migration are both profound life transitions, often involving loss, disruption, and the reorientation of roles, resources, and life priorities. When combined, they may intensify challenges related to health, communication, family relationships, and access to care.^6^ Research across Europe shows that older foreign-born adults, on average, face higher risks of ill health,^7,8^ economic vulnerability,^8,9^ social exclusion,^10^ and lower subjective wellbeing.^11^ In Sweden, research has mainly focused on such structural inequalities and on patterns of service use.^9,12,13^ While essential, this body of work offers limited insight into how older foreign-born adults themselves understand ageing or make sense of what it means to live with dignity.

The present study emerged from a broader doctoral research project exploring perceptions and experiences of ageing among older foreign-born adults in Sweden using a grounded theory approach. In an initial study,^14^ participants’ main concerns centred on three interrelated themes: the importance of family, the value of social connection, and fear of losing independence through physical or cognitive decline. Together, these concerns pointed towards dignity as a unifying ethical concept through which autonomy, relational dependence, and vulnerability could be understood across the ageing process.

Existing research on dignity in later life has emphasised autonomy, recognition, respectful care, and the preservation of personhood, particularly in institutional settings.^15–19^ However, relatively few studies^16–18^ have examined dignity from the perspective of older adults themselves, and even fewer^19^ have addressed the experiences of older migrants. Important ethical questions therefore remain about how dignity is understood, prioritised, and sustained when ageing unfolds in contexts marked by minority positioning, migration, and changing forms of social recognition.

In Nordic health and social care, philosophical frameworks such as Nordenfelt’s model of dignity^20^ have been influential in distinguishing between dignity of merit, moral stature, identity, and the inherent dignity of all human beings. Although highly relevant to eldercare ethics, such frameworks have rarely been explored in relation to older migrants’ lived experiences, where dignity may be negotiated through language, care relations, family expectations, and belonging in institutions not shaped by one’s earlier life world. At the same time, Sweden’s commitment to equality in care is often operationalised through standardised practices. While intended to ensure fairness, standardisation may sit uneasily alongside person-centred care, which requires attention to biography, values, and ways of making meaning. Migration may render this tension especially visible, as communicative barriers, altered family roles, and experiences of cultural displacement can complicate how dignity is recognised and safeguarded in everyday care.

Against this background, the aim of the present study is to explore how older foreign-born adults in Sweden perceive and articulate dignity while ageing. By centring their voices and lived experiences, the study seeks to deepen ethical understanding of dignity as a relational, dynamic, and contextually negotiated phenomenon, and to inform ethically grounded care in an increasingly diverse ageing population.

## Methodology

### Study design and setting

This study employed a qualitative design guided by the Glaserian paradigm of Grounded Theory,^21^ which facilitates the systematic generation of theory through iterative data collection and analysis. The research was conducted in a medium-sized municipality in south-eastern Sweden, with a foreign-born population broadly reflecting national patterns in both proportion and composition.

### Participants

Focus groups were selected as the primary data collection method because they allow participants to build on one another’s reflections and generate rich accounts of complex topics such as dignity within a limited timeframe. To promote open dialogue and linguistic comfort, particularly for older foreign-born adults with limited Swedish proficiency, participants were organised into language-specific groups: one Chinese-, one Finnish-, two Arabic-, and one Spanish-speaking focus group. This strategy aimed to capture variation across larger and smaller immigrant-language groups.

Eligibility criteria were being foreign-born, aged 60 years or older, and able to participate independently in conversation.

Following the Chinese focus group, an individual interview was conducted with an 81-year-old Chinese-speaking participant (C5) who declined the group format and preferred a one-to-one conversation. This interview was retained because the participant represented an older, analytically informative case with rich experience of ageing and caregiving.

Recognising heterogeneity in immigration histories and socioeconomic circumstances within Sweden’s large Arabic-speaking population, the two Arabic-speaking groups were purposefully differentiated by level of integration (Swedish-speaking vs non-Swedish-speaking participants).

Small focus groups are methodologically appropriate when participants have specialised experience and are hard to reach, or when topics are potentially sensitive.^22–24^ Given language-related barriers and recruitment challenges among older foreign-born adults, this study used mini-focus groups^24^, p. 17 of three to four participants to support participation and psychological safety. One Spanish-speaking participant withdrew immediately before the session, resulting in a final group of three.

In total, 23 individuals participated. Participant characteristics and data collection context are presented in Table 1, in the order in which interviews were conducted.

**Table 1.** Participant characteristics and data collection context.

Participant	Age (years)	Gender	Age at migration to Sweden (years)	Country of birth
Chinese-speaking focus group (conducted in Mandarin)				
C1	69	Female	31	China
C2	63	Male	23	China
C3	62	Female	25	China
C4	66	Female	48	China
Individual interview (conducted in Mandarin)				
C5	81	Female	35	China
Arabic-speaking focus group 1 (conducted in Swedish)				
A1	62	Male	45	Iraq
A2	62	Male	45	Iraq
A3	62	Female	46	Iraq
A4	64	Female	19	Syria
A5	60	Female	13	Turkey
A6	60	Male	37	Iraq
Finnish-speaking focus group (conducted in Swedish)				
F1	62	Female	6	Finland
F2	85	Female	30	Finland
F3	85	Male	30	Finland
F4	69	Female	19	Finland
F5	78	Female	29	Finland
Arabic-speaking focus group 2 (conducted with Swedish–Arabic interpreter)				
A7	64	Female	45	Iraq
A8	68	Female	60	Iraq
A9	64	Male	47	Iraq
A10	67	Male	49	Iraq
Spanish-speaking focus group (conducted in Swedish)				
S1	67	Female	24	Chile
S2	76	Male	39	Chile
S3	68	Female	22	Chile

### Recruitment

A multifaceted recruitment strategy was used to reach this often ‘hard-to-reach’ population. Initial visits to eight municipal social centres, where older adults commonly gather, revealed few older adults of non-European origin, highlighting the limitations of this recruitment channel.

Subsequently, collaboration with three community organisations facilitated recruitment for the Finnish- and Arabic-speaking groups. While this strategy may have introduced selection bias towards more socially connected individuals, it represented a deliberate trade-off to enhance trust, accessibility, and participant comfort.

Participants for the Chinese- and Spanish-speaking groups were recruited through convenience sampling followed by snowball sampling.^25^

### Data collection

Data consisted of five focus group discussions and one individual interview conducted by the first author between October 2024 and February 2025. All sessions were guided by a single open-ended question: ‘What does dignity mean for you while ageing?’ Discussions lasted 45–60 min.

The first author, a native Mandarin speaker and authorised interpreter, conducted the Chinese-language sessions in Mandarin. The remaining sessions were conducted in Swedish, with an authorised Arabic interpreter assisting in the second Arabic-speaking focus group. Although interpreter-mediated data collection may influence what is shared and how meaning is conveyed,^26,27^ we judged that including participants who might otherwise have been excluded outweighed these limitations. All audio recordings were transcribed verbatim in Swedish; the Mandarin interviews were translated during transcription by the first author.

Analysis and sampling proceeded iteratively. The Chinese-speaking focus group generated the preliminary conceptual structure, which was subsequently elaborated across later groups; the individual interview served as a supplementary comparative source. After the Chinese-, first Arabic-, and Finnish-speaking groups, the core category and its major relationships had become analytically clear. Later sampling was therefore theoretical: the second Arabic-speaking group explored variation by level of integration, and the Spanish-speaking group was used to elaborate remaining conceptual gaps. These later data enriched the existing categories but did not generate new main categories or substantively alter the emerging theory. Theoretical saturation was thus judged at the level of the core category and major category properties.

### Ethical considerations

Ethical approval was granted by the Swedish Ethical Review Authority (Dnr: 2023-04950-01). Participants received written and oral information about the study and provided written informed consent before data collection. Confidentiality was ensured through the use of participant codes.

### Data analysis

Analysis followed the constant comparative method central to Grounded Theory and proceeded iteratively through coding, constant comparison, memo-writing, and theoretical integration. Transcripts were coded line by line, and codes were compared within and across interviews to refine concepts and develop broader categories. Within-group interaction, including confirmation and qualification, was also treated as analytic data. Extensive memo-writing was used to examine category properties and relationships and contributed to identifying the core category. Once the core category and its relationships to the major categories had become analytically stable, the emerging theory was compared with relevant literature. At this stage, Bakan’s agency/communion framework^28^ was introduced as a sensitising interpretive resource to deepen understanding of the tension between control and connection.

The first author, a doctoral student, conducted the study design, recruitment, data collection, transcription management, initial coding, memo-writing, and drafting of the manuscript. The co-authors, who were also the first author’s doctoral supervisors, were senior researchers and functioned as an analytic discussion group throughout the study. Through regular meetings, they discussed emerging codes, challenged interpretations, and refined the developing conceptual structure, including theoretical sampling decisions. Their complementary experience in gerontology, caring science, and qualitative methodology supported critical reflection during the analysis.

## Findings

This study shows that dignity in later life was experienced not as a stable attribute or externally granted condition but as a dynamic, lifelong negotiation. Across narratives, dignity emerged through balancing two fundamental concerns: control, understood as autonomy, agency, and continuity of self; and connection, understood as relational belonging, care, and recognition by others. Dignity was described as fragile and hard-won, sustained only as long as individuals could negotiate between these concerns under changing life conditions.

The analysis generated one core category – Living and Dying in Dignity: A Lifelong Negotiation of Control and Connection – and four interrelated categories: The Prerequisites of Dignity, Maintaining Dignity in Everyday Life, The Art of Letting Go, and Surrendering with Trust. These were not discrete stages but recurring orientations within a shared trajectory. Participants often began by asserting control over everyday life. As ageing, illness, or dependency progressed, this stance was recalibrated through adaptation and acceptance and could culminate in surrender grounded in trust. Movement between these orientations was neither linear nor uniform, but shaped by health, relationships, migration experiences, and existential outlooks.

### The prerequisites of dignity

This category identifies the conditions participants viewed as necessary for dignity in later life, outlining when dignity could be personally experienced and defended – or, when agency diminished, ethically upheld by others.

#### The cognitive threshold

Participants consistently described dignity as contingent on an intact sense of self, rooted in cognitive capacity and relational recognition. The ability to think, judge, remember, and be recognised by others was framed as a non-negotiable basis of dignity. Several participants described what can be understood as a cognitive threshold, beyond which dignity could no longer be personally experienced. When reflecting on dementia and loss of cognitive selfhood, one participant said:

‘For my own part, I see no dignity or value if I have lost my cognitive ability to make judgments. What does it matter when I don’t know who I am?’ (C2)

This concern extended beyond internal awareness to mutual recognition. No longer knowing – or being known by – significant others was described as dissolving the very relationship through which dignity is affirmed. When discussing the limits of marital caregiving under cognitive decline, one participant explained:

‘I say this to my husband: As long as you are clear in your head and recognize me, I will take care of you… But if one day you no longer recognize me, then it’s better you move to a nursing home’. (C4)

#### Autonomy as cornerstone

Autonomy emerged as the active expression of selfhood and a central marker of dignity as long as cognitive capacity remained intact. Participants repeatedly emphasised retaining decision-making authority over their own lives. They were particularly concerned about situations in which others – both healthcare professionals and younger family members – tried to ‘help’ by speaking over them or deciding on their behalf. Such actions were experienced not as care but as a denial of their status as rational and morally competent persons.

‘The most important thing for me is to decide over my own life. No one gets to decide over me, not even my family’. (A5)

#### Connection as safeguard

Participants distinguished between dignity as personally experienced and dignity as ethically owed. When cognitive selfhood was perceived as compromised, dignity was no longer understood as something the person could actively enact. Instead, it became dependent on the ethical conduct of others.

In these situations, care was framed as an expression of love, responsibility, and moral duty enacted by family members, professionals, and society, rather than as an experience of personal dignity for the individual who could no longer recognise or claim it:

‘When the intellect is gone, the dignity of the older person is up to the hospitals, the nursing homes, and the relatives. There is nothing the elderly person can do to defend it anymore’. (C5)

Dignity thus shifted from being personally enacted to being relationally upheld. Beyond the cognitive threshold, connection became the safeguard of dignity.

### Maintaining dignity in everyday life

This category moves from foundations to the everyday work of sustaining dignity through managing time and perspective, safeguarding autonomy in the home, and maintaining meaningful relationships and activities.

#### The sovereignty of ‘now’

Participants described a range of strategies for preserving selfhood and control in the present, thereby buffering an uncertain future. Across groups, ‘living in the moment’ emerged as a deliberate stance grounded in the recognition that the present was the only temporal domain over which they still retained agency:

‘I never think about questions like what a dignified life and dignified death are. I just take one day at a time… Then we’ll see!’ (F5)

Living in the present was not framed as denial but as an active reorientation of values. For many, it also reflected a sense of moral completion: having fulfilled responsibilities towards children, family, and society, later life was now a legitimate time to rest, enjoy, and prioritise themselves. This was closely tied to trust in family and in the Swedish welfare system, which reduced anxiety about future dependency, finances, and care.

#### Home as locus of identity

Home emerged as a central site for dignity, functioning as both a physical and symbolic space of autonomy, identity, and continuity. Remaining at home enabled participants to preserve everyday rhythms, habitual practices, language use, and a sense of authorship over daily life. Linguistic familiarity within the home was described as especially important when cognitive capacity began to decline:

‘To remain in one’s own home for as long as possible… For someone like my mother who has memory issues, it is even more important that there is someone who talks to her, in her language, Finnish’. (F1)

Home was also described as a space of peaceful independence and freedom, often contrasted with institutional settings perceived as governed by routines that could override personal preferences and identities:

‘I am a happy man! I fix my own things at home… No one nags me, and I nag no one’. (S2)

Across these accounts, home was not merely a preferred residence but an existential anchor where individuals could remain recognisable to themselves and others.

#### Meaningful engagements

Dignity was further sustained through meaningful engagements that connected individuals to purpose, rhythm, and recognition. These ranged from solitary, self-chosen activities to companionship with humans and non-humans, and were described as protections against isolation, passivity, and existential emptiness:

‘To have some occupation - for me, that is being out in nature. In the forest I find everything I need … I also have a cat – she is my best friend’. (F3)

Participants emphasised that dignity depended not on the quantity of social ties but on access to forms of connection that felt meaningful and self-congruent. Community therefore took diverse forms beyond the nuclear or extended family. Some valued close everyday ties with neighbours, while others emphasised a more episodic but symbolically important solidarity within their communities:

‘We Chileans in Sweden don’t meet as often anymore, but if something happens back home or a fellow Chilean faces hardship, we are still here when it matters’. (S3)

Across these accounts, meaningful engagement helped individuals remain socially visible, emotionally connected, and anchored in a responsive world.

### The art of letting go

This category captures a pivotal phase in the dignity negotiation: the point at which maintaining full control was no longer possible and dignity had to be renegotiated through adaptation, acceptance, and moral boundary-setting.

#### Reconciling ideals with new realities

Across groups, participants described a pragmatic acceptance of ageing-related losses, including physical decline, changing family roles, and altered social expectations. This was not experienced as defeat but as an ethical adjustment to changing circumstances.

A recurring theme concerned changing expectations of family care. Participants did not express entitlement to intergenerational caregiving, but instead reformulated care in ways that fitted contemporary realities. In reflecting on changed expectations of intergenerational care after migration, one participant said:

‘Our children still care about us. It’s just that they care about us in some other ways’. (S1)

Here, dignity was preserved not by insisting on past norms but by redefining expectations in ways that protected self-respect and relational balance. Letting go thus prevented resentment, dependency, or moral rupture within families.

Participants also described deeper existential work aimed at reconciling bodily decline and mortality. Letting go involved cultivating acceptance, gratitude, and forgiveness:

‘The ageing has started to be felt in the body every day… But I am grateful for every day that passes. You just have to resign yourself to this new path and adapt your lifestyle to it’. (C1)

‘And then, what is it that one cannot let go of? What is it that one cannot forgive?’ (C2)

In these accounts, letting go functioned as a form of moral and existential maturation that sustained dignity despite loss.

#### Weighing life against suffering

A particularly charged dimension concerned reflections on suffering and the limits of a life worth living. While participants broadly accepted ageing and decline as inevitable, they distinguished between living with limitations and living in a state of perceived meaningless suffering. For some, letting go meant articulating a boundary beyond which life was no longer experienced as dignified:

‘I do not want to live long if I become bedridden… I do not want to be dependent’. (A10)

Such statements reflected an effort to reclaim moral agency in the face of anticipated vulnerability.

Religious and spiritual framing was most visible in the Arabic-speaking groups, especially among participants with shorter residence in Sweden, where suffering and death were more often interpreted in relation to God, destiny, and endurance:

‘We humans must have patience, as Jesus endured crucifixion and was resurrected. That is what suffering can lead to’. (A9)

Here, dignity was not necessarily lost through suffering but could be transformed by it. Letting go was therefore not a single pathway, but a morally complex negotiation shaped by personal values, faith, and ideas of what makes life – and its end – worth living.

### Surrendering with trust

This final category captures a potential culmination of the dignity negotiation: a conscious surrender of control grounded in trust. At this stage, dignity was no longer primarily sustained through personal agency, but through confidence that one would be cared for, recognised, and held within reliable relational, institutional, or existential support.

#### Trusting the human safeguard

For many participants, surrender became possible through trust in others, but the location of that trust differed across groups. Chinese-speaking participants placed greatest trust in family and close friends. Finnish- and Spanish-speaking participants more often described emotional closeness with family alongside practical reliance on the welfare state. Arabic-speaking participants emphasised strong family and communal support while also expressing trust in Swedish health and social care as professional safeguards. When reflecting on how institutional care could still preserve dignity, one participant said:

‘Sweden has a very good model for palliative care… they always have time to sit and talk, answer questions, and give the care that is needed’. (A5)

Surrender to institutional care was, however, rarely unconditional. Across all groups, participants stressed that safety and physical care alone were insufficient for dignity. What mattered equally was being recognised in one’s language, habits, values, and cultural ways of living. The wish for culturally adapted eldercare was thus widely shared, although it was expressed differently across groups. For Finnish-speaking participants, this need was described in relation to existing Finnish-language eldercare arrangements in Sweden, which illustrated how linguistic and cultural continuity could support dignity in later life. For other groups, the same need was voiced more as an absence or future aspiration. In discussing what was missing from existing institutional care, one participant said:

‘We must start working on a Chinese-speaking nursing home in this city in the future!’ (C4)

Across these accounts, dignity at this stage depended not only on being cared for but on being understood as oneself.

#### Readiness for the end of life

Surrender also extended to reflections on death, where participants articulated different forms of readiness. The two Arabic-speaking groups differed less in core concern than in framing: the more Swedish-integrated group described adapting to a more secular Swedish way of speaking about death and dying, whereas the less integrated group more explicitly emphasised faith, transcendence, and prior experiences of suffering.

‘We are not afraid of death, because we believe in God’. (A9)

By contrast, Finnish-speaking participants more often emphasised practical preparation and reducing burden on others as a final act of care. When reflecting on readiness for the end of life, one participant said:

‘You can talk to your relatives about your own death… so that they don’t have worries’. (F1)

Chinese-speaking participants more often framed death in philosophical terms, seeing it as a natural return rather than an event requiring control:

‘Death itself is simple, it is to go back into nature’. (C1)

Across these narratives, surrender emerged not as passive loss of control but as an active and meaning-laden orientation towards the end of life. Dignity was sustained through trusting relationships, reliable care structures, and coherent existential frameworks that allowed individuals to let go without fear of abandonment.

Gender did not emerge as a consistent analytic axis. However, in the Arabic-speaking groups, men more often linked dignity to social misrecognition and labour-market exclusion, whereas women more often emphasised trust and relational support. These differences did not alter the core category. Participant age likewise did not produce clearly differentiated accounts, although the older individual interview offered particularly vivid reflections on the cognitive threshold and end-of-life control.

## Discussion

This study contributes to nursing ethics by showing that dignity in later life is neither a fixed state nor an individual possession, but a lifelong negotiation between control and connection. For older foreign-born adults, this negotiation was reshaped by migration-related conditions, especially language, home, family relations, and institutional encounters. The findings therefore suggest that dignity is not best understood as a static principle to be protected in the abstract but as a relational and context-sensitive process that must be actively sustained in everyday life and care.

Participants often spoke of dignity through a language of control, self-determination, and not being spoken over. At first glance, this may appear close to an individualistic model of autonomy. However, autonomy was rarely imagined as independence from others. Rather, it was negotiated within family ties, moral obligations, trust in care, language, and conditions of vulnerability. A relational understanding of autonomy therefore captures our data better than a narrow decisional model. Likewise, dignity emerged not as a culturally identical or philosophically settled concept but as a widely shared ethical concern whose practical meanings were shaped by relational, cultural, and migratory contexts.

The concept of negotiation is central to the theoretical contribution of this study. Negotiation did not refer to a single decision or compromise but to an ongoing recalibration through which dignity was sustained under changing conditions. Participants continually negotiated how much control could be retained, how much dependence could be accepted, which connections remained dignity-preserving, and when surrender became more dignified than continued struggle. This process was practical, moral, relational, and existential at once.

The emergent pattern resonated with Bakan’s distinction between agency and communion.^28^ Participants’ emphasis on autonomy, self-determination, and control reflected agency, while their emphasis on family, relational continuity, and being upheld by others reflected communion. These did not appear as opposites to be resolved once and for all, but as poles continuously negotiated in the pursuit of dignity in later life. Dignity was thus experienced neither as independence alone nor as connection alone, but as the capacity to rebalance these dimensions as life conditions changed.

The ‘cognitive threshold’ was one of the most ethically charged findings. In participants’ accounts, the feared threshold referred not simply to memory loss but to a point at which judgement, self-recognition, and the ability to defend one’s own dignity were perceived to weaken. This places the finding close to ethical debates in dementia care, where dignity and personhood are understood not as exhausted by cognitive performance alone but as shaped by relationships, recognition, and the care environment.^29–31^ What participants feared was therefore not cognitive decline in itself but the possibility that, beyond a certain threshold, dignity would depend on whether others could recognise, interpret, and protect the person they had been and the values by which they wished to live and die.

The findings also provide empirical elaboration of Nordenfelt’s model of dignity.^20^ Most prominently, dignity of identity emerged as non-negotiable, grounded in coherence, recognition, and intact cognitive selfhood. At the same time, participants’ reflections on acceptance, gratitude, and forgiveness suggest a form of moral maturity developed across the life course. A key ethical implication concerns *Menschenwürde*, the inherent dignity of all human beings: once agency weakened, dignity had to be upheld by others. In this sense, dignity became relationally enacted, and families, caregivers, and institutions functioned as moral safeguards for a dignity the individual could no longer actively defend.

While the findings broadly confirm earlier research on dignity in later life, they also show how migration reshapes the contexts in which dignity is negotiated. Language became ethically significant where recognition, familiarity, and continuity of self were at stake, particularly in situations of dependency, cognitive decline, or institutional care. Home likewise assumed heightened significance as a culturally sustained micro-world in which routines, language, and identity remained coherent. Institutionalisation therefore risked not only loss of autonomy, but erosion of the primary setting in which selfhood remained legible.

Migration also reshaped family relations. Expectations of intergenerational care were renegotiated in light of Swedish norms of independence and welfare provision, adding moral and emotional complexity to decisions about autonomy, dependency, and care. At the same time, participants did not portray themselves merely as vulnerable. Their everyday efforts to sustain dignity reflected resilience, adaptation, and meaning-making rather than passive loss. Migration did not diminish moral agency but reconfigured the conditions under which dignity was negotiated.

These reconfigured conditions also shaped how participants understood suffering, endurance, and the moral limits of a life worth living. From a nursing ethics perspective, this challenges end-of-life approaches that rely mainly on abstract criteria such as autonomy or symptom burden detached from context. A dignity-based ethic cannot be reduced either to minimising suffering or to autonomy understood in narrowly individualistic terms. Rather, it requires attentiveness to how dignity is defined, sustained, or threatened within the person’s own moral, relational, and cultural horizon. The ethical question becomes not only whether suffering should be reduced, but what in a person’s remaining life would preserve meaning, coherence, and relational integrity.

## Methodological considerations and limitations

The first author occupied a shifting positionality across the study rather than a fixed insider or outsider role. In the Chinese-speaking group, shared first language and cultural familiarity may have supported trust and depth while also risking taken-for-granted understandings. In the Swedish-speaking and interpreter-mediated groups, greater linguistic distance required more explicit negotiation of meaning and may have shaped both what participants elaborated and what remained less easily conveyed. Interpreter mediation may also have influenced how idiomatic meanings, emotional tone, and interactional dynamics were rendered. Likewise, the Mandarin-language interviews were translated into Swedish during transcription by the first author, preserving closeness to the original speech while also concentrating interviewing, translation, and primary analysis in one researcher. These positional and translational dynamics may have shaped how dignity, as a sensitive and culturally embedded topic, was articulated and interpreted, and were reflected on through memo-writing and analytic discussions.

Recruitment through cultural organisations may have favoured socially connected individuals, potentially underrepresenting more isolated older adults. The individual interview differed from the focus groups in lacking the interactional and co-constructed character of group discussion, and its inclusion in an otherwise focus group-based design introduced a different narrative form. The mini-focus-group format may also have reduced interactional complexity and limited the visibility of disagreement or co-constructed meanings; the findings should therefore be understood as grounded in both within-group interaction and comparison across groups. As the findings are grounded in the Swedish welfare context, transferability to other care systems should be approached cautiously.

## Conclusion and implications

This study shows that dignity in later life is not a fixed condition but a lifelong negotiation between control and connection. For older foreign-born adults, this negotiation is reshaped by migration-related transitions, making recognition, a sense of home, and continuity in relationships especially significant.

The findings suggest that dignity-supporting care requires more than respect for decisional autonomy or physical comfort. In care planning, professionals should explore how the person understands control, which relationships sustain dignity, what makes home meaningful, and what forms of dependence are experienced as tolerable or threatening. Particular attention should be paid to communication in the person’s preferred language where possible, especially in situations of cognitive decline, institutional care, and end-of-life decision-making. The findings also support culturally responsive advance care planning that includes family roles, existential or religious frameworks, and preferences for how dignity should be upheld if the person can no longer defend it independently.

These implications arise from a Swedish welfare-state context and should be transferred cautiously to other settings.

## Data Availability

The datasets generated and analysed during the current study are not publicly available due to the nature of ethical approval, but anonymised data are available from the corresponding author on reasonable requests.*
